# Distribution and classification of the extracellular matrix in the olfactory bulb

**DOI:** 10.1007/s00429-019-02010-8

**Published:** 2019-12-19

**Authors:** Andrea Hunyadi, Botond Gaál, Clara Matesz, Zoltan Meszar, Markus Morawski, Katja Reimann, David Lendvai, Alan Alpar, Ildikó Wéber, Éva Rácz

**Affiliations:** 1 Department of Anatomy, Histology and Embryology, Faculty of Medicine, University of Debrecen, Nagyerdei krt. 98., Debrecen, 4032 Hungary; 2 Division of Oral Anatomy, Faculty of Dentistry, University of Debrecen, Nagyerdei krt. 98., Debrecen, 4032 Hungary; 3 MTA-DE Neuroscience Research Group, Nagyerdei krt. 98., Debrecen, 4032 Hungary; 4 Paul-Flechsig-Institute of Brain Research, Medical Faculty, University of Leipzig, Leipzig, Germany; 5 Department of Anatomy, Histology, and Embryology, Semmelweis University, Budapest, 1085 Hungary; 6 SE NAP Research Group of Experimental Neuroanatomy and Developmental Biology, Semmelweis University, Budapest, 1085 Hungary

**Keywords:** Neural plasticity, Perineuronal net, Hyaluronan, Chondroitin sulfate proteoglycans, Tenascin-R, Link protein

## Abstract

Extracellular matrix (ECM) became an important player over the last few decades when studying the plasticity and regeneration of the central nervous system. In spite of the established role of ECM in these processes throughout the central nervous system (CNS), only few papers were published on the ECM of the olfactory system, which shows a lifelong plasticity, synaptic remodeling and postnatal neurogenesis. In the present study, we have described the localization and organization of major ECM molecules, the hyaluronan, the lecticans, tenascin-R and HAPLN1 link protein in the olfactory bulb (OB) of the rat. We detected all of these molecules in the OB showing differences in the molecular composition, staining intensity, and organization of ECM between the layers and in some cases within a single layer. One of the striking features of ECM staining pattern in the OB was that the reactions are shown dominantly in the neuropil, the PNNs were found rarely and they exhibited thin or diffuse appearance Similar organization was shown in human and mice samples. As the PNN limits the neural plasticity, its rare appearance may be related to the high degree of plasticity in the OB.

## Introduction

**Fig. 1:**
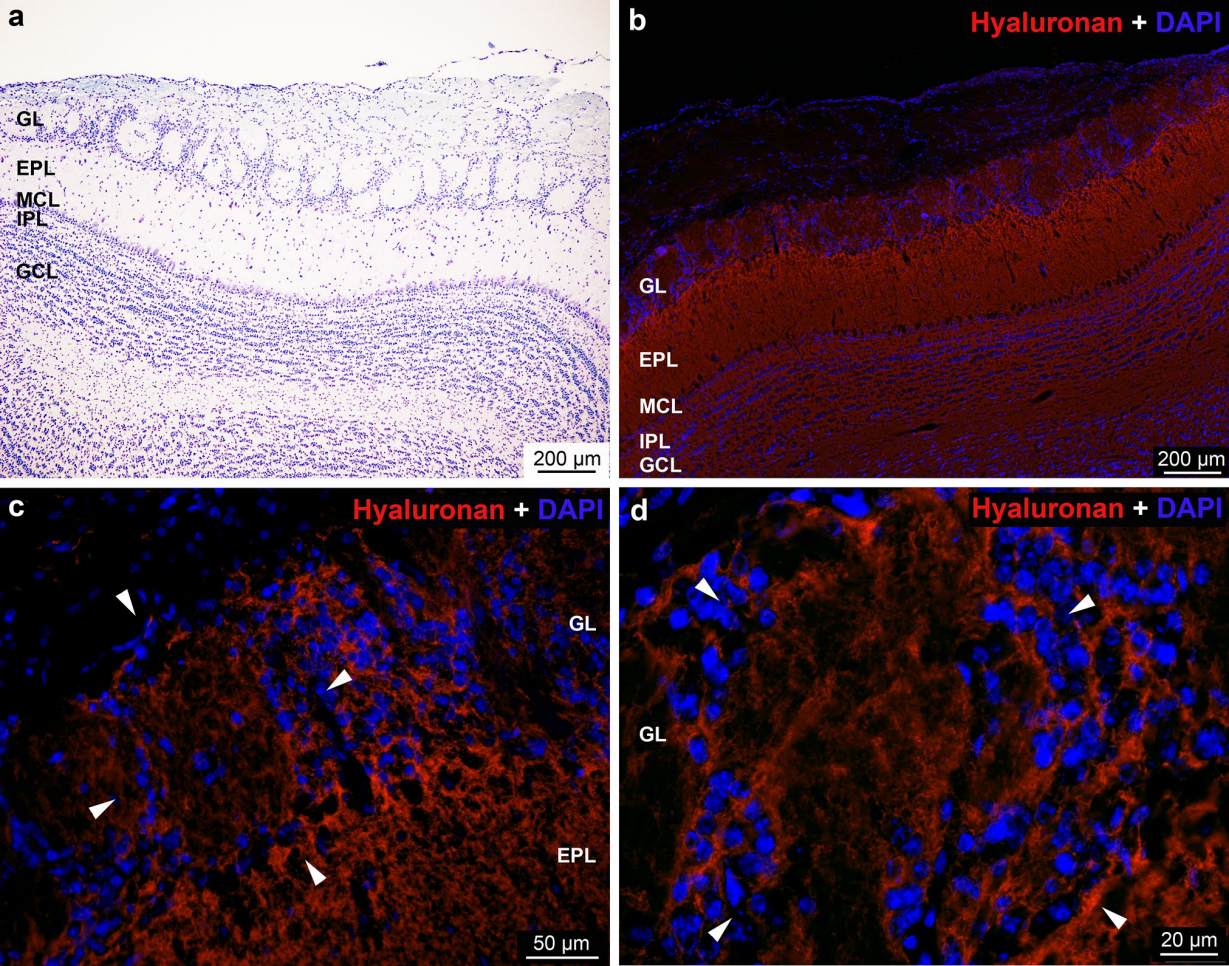
**a** Nissl stained section showing the layers of olfactory bulb (OB) in rat. *GL* glomerular layer, *EPL* external plexiform layer, *MCL* mitral cell layer, *IPL* internal plexiform layer, *GCL* granular cell layer. These abbreviations are applied to the rest of figures*.***b**–**d** Distribution of *hyaluronan* reaction in the rat OB. Nuclei were stained with DAPI. Arrowheads in **c** and **d** show the periglomerular area

The olfactory system, by monitoring odorant molecules of the environment, influences social and sexual behavior. Sensory neurons in the olfactory epithelium express various odorant receptors and the central processes of these cells terminate in the glomeruli of the olfactory bulb (OB). In the OB, several types of interneurons establish complex neuronal networks with the efferent neurons which process the information to the olfactory cortex. The olfactory bulb is divided into multiple layers (Fig. 1a) and the distinct layers have different neuron types categorized conventionally on the basis of localization of cell bodies (Allison 1953; Kosaka et al. 1998; Nagayama et al. 2014; Pinching and Powell 1971). The first set of neurons are located in the glomerular layer (GL), referred to as juxtaglomerular cells (JG) (Kosaka and Kosaka 2011, 2016; Nagayama et al. 2014; Wachowiak and Shipley 2006). The JG cells are further categorized into periglomerular, superficial short axon cells and external tufted neurons (ET). Although, in the olfactory bulb there are some interneurons that do send their axons to extrabulbar areas of the brain (Eyre et al. 2008; Brunjes et al. 2005), the JG interneurons (including periglomerular cells and superficial short axon cells), located in the juxtaglomerular region of the glomerular layer, do not project to the anterior olfactory nucleus. The external plexiform- (EPL) and mitral cell layers (MC) contain mostly the somata of various tufted and mitral cells, which are the major projection neurons of the olfactory bulb. In addition, several subtypes of interneurons are also found in these layers. The internal plexiform layer (IPL) contains the axons of mitral and tufted cells with some of the dendrites of the granule cells. The granule cell layer (GCL) is mostly populated by morphologically heterogeneous interneurons, the granule cells. Several other neurons of the OB have not been classified into these categories and new types of neurons were recently discovered (Merkle et al. 2014; Nagayama et al. 2014).

The neuronal network of the olfactory bulb is continuously reorganized throughout life using two mechanisms. The olfactory sensory neurons have a continuous turnover and their ingrowing axons integrate into the existing neuronal assembly of the glomerulus containing axons expressing the same olfactory receptor genes (Costanzo 2005; Lledo and Saghatelyan 2005; Ma et al. 2017). On the other hand, the structure of the olfactory neuronal network is also modified by the incorporation of newborn neurons arriving from the subventricular zone (Luskin 1993). These processes result in a high degree of neural plasticity, however, the underlying molecular mechanism is not fully understood. It has become clear that molecules of the extracellular matrix (ECM) play an important role in neural development, proliferation, migration, axonal guidance, synapse formation and remodeling (Barros et al. 2011; Bruckner et al. 2008; Celio et al. 1998; Dityatev and Fellin 2008; Dzyubenko et al. 2016; Faissner et al. 2010; Fawcett 2015; Kwok et al. 2011; Reinhard et al. 2015; Wiese and Faissner 2015). The major components of the ECM are (1) hyaluronan (HA), (2) chondroitin sulfate proteoglycans (CSPG) or lecticans including aggrecan, brevican, neurocan, versican (3) glycoproteins e.g., tenascin-R (TN-R), and link proteins (Carulli et al. 2006; Delpech et al. 1989; Dityatev and Schachner 2003; Eggli et al. 1992; Gong et al. 1994; Hartig et al. 1992; Margolis et al. 1975; Matesz et al. 2005; Meszar et al. 2008; Morawski et al. 2012; Szigeti et al. 2006; Yasuhara et al. 1994; Zimmermann and Dours-Zimmermann 2008). In the olfactory bulb only the role of TN-R was examined. TN-R is known to modulate the adult neurogenesis in adult mice but this effect is missing during the embryonic period due to the initiation of TN-R expression during the first postnatal week (David et al. 2013; Saghatelyan et al. 2004). To understand the possible contribution of the other components of the ECM in the plasticity of the olfactory system detailed knowledge on their distribution is required. Therefore, the aim of the present study is to describe the molecular composition and organization of these ECM molecules in various layers of the olfactory bulb in the rat. Here, we particularly focus on condensed forms of ECM, the perineuronal net (PNN), axonal coat, and nodal ECM, which gain their definitive molecular and structural organization postnatally by the time of stabilization of synaptic contacts and completion of myelination (Oohashi et al. 2015). In the case of some molecules, we extended the study to human and mices samples.

## Materials and methods

### Animals and tissue processing in rat

The study protocol was carried out in accordance with the guidelines of the Animal Care Committee of the University of Debrecen, Debrecen, Hungary and the national laws and EU regulations (license number: 6/2017/DEMAB).

Adult male (12–14-week old) Wistar rats (*n* = 5) from Charles River Laboratory (Strain Crl:WI), weighting from 250 to 300 g were deeply anesthetized with intraperitoneal injection of 10% urethane (1.3 ml/100 g body weight; Reanal, Budapest, Hungary) and perfused transcardially with physiological saline. After removal of the olfactory bulbs, they were immersed into Sainte-Marie’s fixative (99% absolute ethanol and 1% glacial acetic acid) for one day at 4 °C. The specimens were embedded in paraffin and cross sections were made with microtome at a thickness of 8 μm. The sections were collected on silane coated slides and left to dry overnight at 37 °C. After deparaffination, sections were rehydrated and washed in phosphate-buffered saline, pH 7.4 (PBS) and treated with 3% H_2_O_2_ dissolved in bidestilled water for 10 min at room temperature (RT).

### Animals and tissue processing in mice

Experiments were conducted on transgenic mice deficient for the CSPG neurocan (background: C57BL/6 N). The murine neurocan gene has a size of about 25 kb, with the coding sequence for the mRNA distributed over 15 exons (Rauch et al. 1995). To generate the neurocan knock-out mouse line, the portion of the neurocan allele containing the TATA box, transcription start site, exon 1 and exon 2 was replaced with a loxP neomycin thymidine kinase loxP cassette. As a result, the transcription of neurocans is prevented. The transgenic mice (*ncan*^*−/−*^) are viable, fertile and show no apparent abnormalities (phenotype and behavior).

Both homozygous knockout mice (*ncan*^*−/−*^) and wild type littermates (*ncan*^+*/*+^) derived from heterozygous parents. The animals were housed in the animal care facilities of the Experimental Center of the Faculty of Medicine, University of Leipzig in a temperature-controlled environment with free access to food and water and 12 h dark/light cycle. The genotype of the experimental animals was determined by PCR. Mice of both sexes were used.

All experiments were carried out in accordance with the German law on the use of the laboratory animals and were approved by the Saxonian District Government, Leipzig (license number: T27/16).

The animals were anesthetized with CO_2_ and perfused transcardially with 0.9% NaCl followed by fixative (4% paraformaldehyde and 0.1% glutaraldehyde). Brains were removed, postfixed overnight, and cryoprotected in 30% sucrose with 0.1% sodium azide before cutting 30 µm thick slices in the frontal plane with a cryomicrotome (Zeiss Hyrax S30 with freezing unit Zeiss Hyrax KS34). Before staining, the slices were treated with 60% methanol and 2% H_2_O_2_ for 1 h, followed by blocking solution (2% BSA—WFA, 2% BSA, 0.3% milk powder and 0.5% donkey serum—aggrecan and brevican) for 30 min.

### Histochemistry and immunohistochemistry in rat and mice

#### Simple fluorescent labeling of ECM molecules in rat

Before the histochemical and immunohistochemical reactions, specimens were blocked for 30 min at RT in 3% bovine serum albumin (BSA) (hyaluronan, HA; *Wisteria floribunda* agglutinin, WFA; versican), 3% BSA + 10% normal goat serum (NGS) (aggrecan), 3% BSA + 10% normal rabbit serum (NRS) (mouse monoclonal anti-chondroitin sulfate proteoglycan, Clone Cat-301; neurocan), 3% BSA + 10% normal donkey serum (NDS) (brevican, TN-R, HAPLN1), all dissolved in PBS.

##### Histochemical reactions

HA was detected using biotinylated Hyaluronan Binding Protein (bHABP; AMS Biotechnology, Abingdon, UK). WFA histochemistry was performed using biotinylated *Wisteria floribunda* agglutinin (bWFA; Sigma-Aldrich, St. Louis, MO, USA), a lectin that binds to *N*-acetylgalactosamine residues of CSPG-glycosaminoglycan chains and glycoproteins, as a marker of PNNs (Giamanco et al. 2010; Hartig et al. 1992). After blocking, sections were incubated in a solution of bHABP or bWFA, both dissolved in PBS containing 1% BSA, overnight at 4 °C. Reactions were visualized by incubating the samples with Streptavidin AlexaFluor 555 (Life Technologies, Carlsbad, CA, USA) for 1 h, diluted in 1:1000, in PBS.

##### Immunohistochemical reactions

After blocking as described above, slides were incubated in the following primary antibodies: rabbit polyclonal anti-aggrecan (Merck Millipore, Billerica, MA, USA), mouse monoclonal anti-chondroitin sulfate proteoglycan (Clone Cat-301, Sigma-Aldrich), mouse monoclonal anti-versican (12C5; Developmental Studies Hybridoma Bank, DSHB, Iowa City, IA, USA), mouse monoclonal anti-neurocan (1F6; DSHB), sheep polyclonal anti-brevican (R&D Systems, Minneapolis, MN, USA), goat polyclonal anti-tenascin-R (R&D Systems), goat polyclonal anti-HAPLN1 (R&D Systems). For better antigen exposure of aggrecan, Cat-301, versican and brevican molecules, sections were digested with chondroitinase ABC (0.02 U/ml; Sigma-Aldrich) in specific Tris sodium-acetate buffer, pH 8 (according to the manufacturer’s instructions) for 1 h at 37 °C.

Primary antibodies were diluted in PBS and 1% BSA + 3% NGS (aggrecan), 1% BSA (versican), 1% BSA + 3% NRS (Cat-301, neurocan), 1% BSA + 3% NDS (brevican, TN-R, HAPLN1), overnight at 4 °C. The incubation was followed by repeated rinsing in PBS. More details of primary reagents are reported in Table 1.

**Table 1:** Probe, lectin and primary antibodies used for detection of ECM molecules in the rat

	bHABP^a^	bWFA^b^	Anti-aggrecan	CAT-301^c^	Anti-versican	Anti-neurocan	Anti-brevican	Anti-tenascin-R	HAPLN1^d^
Supplier, cat. no	AMS Biotechnology; AMS.HKD- BC41	Sigma-Aldrich; L1516	Merck Millipore; AB1031	Sigma-Aldrich; MAB5284	DSHB; 12C5	DSHB; 1F6	R&D Systems; AF4009	R&D Systems; AF3865	R&D Systems; AF2608
Species of origin, type	Recombinant human versican G1 domain expressed in *E. coli*; biotinylated	Lectin isolated from *Wisteria floribunda;* biotinylated	Rabbit; polyclonal, IgG	Mouse; Monoclonal, IgG1	Mouse; monoclonal, IgG1	Mouse; monoclonal, IgG1	Sheep; polyclonal, IgG	Goat; polyclonal, IgG	Goat; polyclonal, IgG
Immunogen	–	–	GST fusion protein containing amino acids 1177–1326 of mouse aggrecan	Feline spinal cord fixed gray matter	Hyaluronate-binding region of human versican	PBS-soluble CSPGs from rat brain	Mouse myeloma cell line NS0 derived recombinant human Brevican Asp23 Pro911	Mouse myloma cell line NS0-derived recombinant human tenascin-R isoform 1, Glu34-Phe1358	Mouse myeloma cell line NS0-derived recombinant human HAPLN1, Asp16- Asn354
Dilution	1:100	1:500	1:500	1:100	1:100	1:100	1:100	1:300	1:300

^a^Biotinylated Hyaluronan Binding Protein

^b^Biotinylated *Wisteria floribunda* agglutinin

^c^Anti-chondroitin sulfate proteoglycan Clone CAT-301

^d^Hyaluronan and Proteoglycan Link Protein 1

The following secondary antibodies were used: goat-anti-rabbit IgG AlexaFluor 555 (Life Technologies) (aggrecan), rabbit-anti-mouse IgG AlexaFluor 555 (Life Technologies) (Cat-301, versican, neurocan), donkey-anti-sheep IgG AlexaFluor 555 (Life Technologies) (brevican), and donkey-anti-goat IgG AlexaFluor 555 (Life Technologies) (TN-R, HAPLN1), for 1 h, each diluted in 1:1000, in PBS.

#### Simple fluorescent labeling of ECM molecules in mice

In mice, the WFA, aggrecan and brevican stainings were almost identical used in rat. The differences were that (1) for rabbit polyclonal anti-aggrecan (1:500, AB 1031, Merck Millipore, Billerica, MA, USA) and mouse anti-brevican (1:100, clone 2/Brevican, BD Transduction Laboratories, Heidelberg, Germany) stainings no chondroitinase ABC treatment was applied, (2) that the primary antibodies were diluted in PBS and 1% BSA + 3% NDS and (3) that the secondary antibodies used were donkey-anti-rabbit IgG Cy3 and donkey-anti-mouse IgG Cy3 (1:1000, 1 h, Sigma-Aldrich).

#### Double fluorescent labeling in rat

Double fluorescent labeling was made using neurofilament or microtubule-associated protein 2 (MAP2) antibodies in combination with specific ECM markers. The following fluorescent labelings were combined: mouse monoclonal anti-neurofilament (Sigma-Aldrich) + WFA, mouse monoclonal anti-neurofilament + aggrecan, rabbit polyclonal anti-MAP2 (Merck Millipore) + Cat-301, rabbit polyclonal anti-neurofilament (Sigma-Aldrich) + versican, mouse monoclonal anti-neurofilament + brevican, rabbit polyclonal anti-MAP2 + brevican, mouse monoclonal anti-neurofilament + HAPLN1, rabbit polyclonal anti-MAP2 + HAPLN1 (Table 2).

**Table 2:** Primary antibodies used for double labeling with ECM molecules in the rat

	Anti-neurofilament	Anti-neurofilament	Anti-MAP2^a^
Supplier, cat. no	Sigma-Aldrich; N4142	Sigma-Aldrich; N5389	Merck Millipore; AB5622
Species of origin, type	Rabbit; polyclonal, IgG	Mouse; monoclonal, IgG1	Rabbit; polyclonal, IgG
Immunogen	Purified neurofilament 200 from bovine spinal cord	Purified neurofilament 200 from pig spinal cord	Purified Microtubule-associated protein from rat brain
Dilution	1:80	1:40	1:500

^a^Microtubule-Associated Protein 2

Prior to the incubation with neurofilament or MAP2 primary antibodies, specimens were blocked for 30 min at RT in 3% BSA + 10% NGS (mouse anti-neurofilament), 3% BSA + 10% NDS (rabbit anti-neurofilament, rabbit anti-MAP2). Primary antibodies were diluted in PBS with 1% BSA + 3% NGS (mouse anti-neurofilament) and 1% BSA + 3% NDS (rabbit anti-neurofilament, rabbit anti-MAP2). Visualization of reactions was by goat anti-mouse IgG AlexaFluor 488 (mouse anti-neurofilament; Life Technologies) or donkey anti-rabbit IgG AlexaFluor 488 (rabbit anti-neurofilament, rabbit anti-MAP2; Life Technologies). Slides were coverslipped with ProLong® Diamond Antifade Mountant with DAPI (Life Technologies).

#### Image acquisition and postprocessing

Images from rat tissue were recorded using Olympus CX31 epifluorescent light microscope with DP27 digital camera and processed by Photoshop CS4 v11.0 (Adobe Systems Inc., San Jose, CA, USA), with minimal adjustments of contrast and background. Higher magnification images were taken with confocal microscope (Olympus, FV-3000) using a 10 × dry objective for panoramic view of the olfactory bulb and then an oil immersion 60 × objective lens (PlanApoN, N.A. 1.40) for acquiring high-resolution confocal stacks. We also applied super-resolution microscopy using FV-OSR module of the FV-3000 software. During acquisition, the confocal and super-resolution images were then postprocessed with spectral deconvolution using the build in software module of the FV-3000 software. Image stacks taken by the super-resolution mode were also post processed with deconvolution (Olympus, CellSens 3D deconvolution module) using adaptive point spread function (PSF).

Images from mouse tissue were recorded using Keyence BZ9000 Biorevo automated epifluorescent light microscope processed by Photoshop CS6 (Adobe Systems Inc., San Jose, CA, USA), with minimal adjustments of contrast and background.

### Human tissue preparation and immunohistochemistry

We have applied direct perfusion via the internal carotid- and vertebral arteries, which facilitated the preservation of tissue integrity relative to alternative fixation methods. Human brains (*n* = 2, gender and age: female/83 years and male/79 years, ethical approval: TUKEB 84/2014, Hungary) were first perfused with physiological saline followed by a fixative containing 2% PFA and 0.1% glutaraldehyde in 0.1 M Tris-buffered saline (TBS, pH 7.4) 7 h or 11 h after death. The removal and subsequent preparation of postmortem human tissues were in accordance with relevant ethical guidelines of Semmelweis University (1998, Budapest, Hungary). Blocks of olfactory bulbs were dissected out, post-fixed in 2% PFA in TBS for 72 h, followed by immersion in cryoprotective 30% sucrose in 0.1 MPB (pH 7.4) overnight. Coronal sections (50 μm) were cut on a cryostat microtome and processed for immunohistochemistry. Free-floating sections were rinsed in PB (pH 7.4) and pre-treated with 0.3% Triton-X 100 (in PB) for 1 h at 22–24 °C to enhance the penetration of antibodies. Non-specific immunoreactivity was suppressed by incubating our specimens in a cocktail of 5% normal donkey serum (NDS; Jackson Immunoresearch, Cambridgeshire, UK), 2% BSA and 0.3% Triton X-100 (Sigma-Aldrich) in PB for 1 h at 22–24 °C.

Sections were exposed (16–72 h at 4 °C) to the primary antibody (goat polyclonal anti-HAPLN1, 1:200, R&D systems or rabbit polyclonal anti-brevican 50 kDa fragment, 1:2000, gift from R. Matthews or mouse monoclonal anti-aggrecan HAG7D4, 1:10, Acris; Herford, Germany) diluted in PB to which 0.1% NDS and 0.3% Triton X-100 had been added. After repeated washing sections were exposed to biotinylated anti-mouse or rabbit or goat IgG raised in donkey (1:1000, Jackson Immunoresearch, for 2 h at 22–24 °C) followed by pre-formed avidin–biotin complexes also incorporating horseradish peroxidase for 1 h at 22–24 °C. Immunosignals were visualized by 3,3′-diaminobenzidine (Sigma-Aldrich, 0.025%) as chromogen intensified with Ni-ammonium sulphate (Merck Millipore, 0.05%) in the presence of 0.001% H_2_O_2_ as substrate (dissolved in 0.05 M Tris buffer, pH 8.0).

Glass-mounted sections were coverslipped with Aquamount embedding medium (Dako North America, Campinteria, CA, USA). Results of chromogenic stainings were captured on an Olympus BX-51 microscope.

For Western Blotting, brains (*n* = 2) were removed with a postmortem delay of 4 or 6 h from the skull, their olfactory bulbs and primary motor cortex excised and snap-frozen. Simultaneously, we have processed olfactory bulbs excised from brains of decapitated 12 week old rats (*n* = 2). Blocks were homogenized in TNE buffer containing 0.5% Triton X-100 (Sigma), 1% octyl-β-D-glucopyranoside (Calbiochem, Merck Millipore), 5 mM NaF, 100 μM Na_3_VO_4_ and a cocktail of protease inhibitors (CompleteTM, Roche, Basel, Switzerland) using an Ultra Turrax® tube drive (IKA, Staufen, Germany). Cell debris and nuclei were pelleted by centrifugation (800 g, 10 min at 4 °C). Protein concentrations were determined by Bradford’s colorimetric method. Samples were diluted to a final protein concentration of 2 μg/μl, denatured in 5 × Laemmli buffer, and analyzed by SDS-PAGE on 8% or 10% resolving gels. After transferring onto Immobilon-FL polyvinylidene difluoride membranes (Millipore), membrane-bound protein samples were blocked in 3% BSA and 0.5% Tween-20 diluted in TBS for 1.5 h and subsequently exposed to anti-HAPLN-1 (1:400), anti-brevican (1:500), anti-aggrecan (AB1031) (1:2000) or anti-tenascin-R (Merck/Millipore) (1:1000) primary antibodies overnight at 4 °C. Appropriate combinations of HRP-conjugated secondary antibodies were used for signal detection (Jackson; from goat, rabbit or mouse hosts; 1:10,000, 2 h). Image acquisition and analysis were performed on a Bio-Rad XRS + imaging platform.

## Results

### Distribution pattern of ECM in the olfactory bulb of the rat

The staining pattern and intensity of the histochemical and immunohistochemical reactions varied in the layers of the OB. The most typical form was a diffuse staining in the neuropil which, depending on the type of reaction contained small, ring-like structures representing the axonal coats (Brückner et al. 2008) and darkly stained dots corresponding to the nodal ECM (Bekku et al. 2009; Bekku and Oohashi 2010). The third form of condensed ECM, the perineuronal net (PNN), which surrounds the cell body and dendrites, is classified into thin, robust or diffuse forms in various parts of the CNS (Wegner et al. 2003; Jager et al. 2013). The PNN was rarely present in the OB and appeared as the thin form.

Using of HA probe, the reaction was detected throughout the OB mostly in the neuropil (Fig. 1b). In the glomerular layer, the periglomerular area showed stronger reactivity (Fig. 1c, d), however, the presence or absence of PNN cannot be identified due to the densely packed cells in this area. In the glomeruli, irregular HA positive and negative patches were shown with higher magnification (Fig. 1c, d). In the other layers of OB, the HA reaction was moderate, except for the intense staining in the outer part of the external plexiform layer (Fig. 1b, c). Occasionally, very thin PNNs were recognizable around a population of the mitral cells (not shown).

**Fig. 2:**
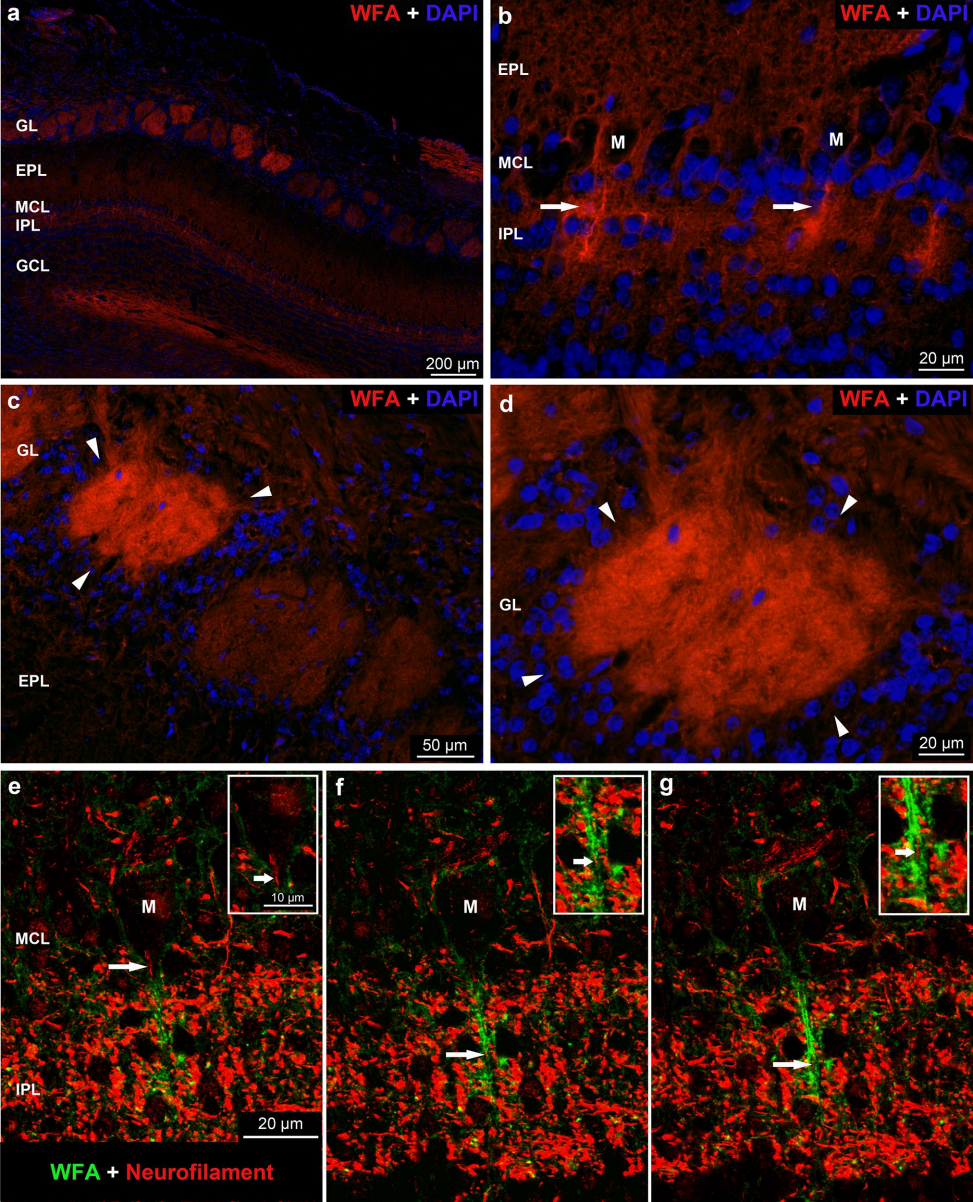
**a** Low magnification image on the distribution of *Wisteria floribunda* agglutinin (WFA) reaction in different layers of the rat OB. **b** Intensely stained WFA positive bands (arrows) are shown in the outer part of the internal plexiform layer (IPL). **c**, **d** Arrowheads label the periglomerular area surrounding heavily stained glomeruli. Nuclei were stained with DAPI (**a**–**d**). **e**–**g** Neurofilament immunoreactivity (red) represents the axon of a mitral cell (arrows) surrounded by very strong WFA positive bands (green) in serial sections. *M* cell body of mitral cells surrounded by perineuronal net (green)

The WFA reaction was detected in the neuropil of the whole OB, showing layer-specific staining pattern and intensity (Fig. 2a). In the glomerular layer, the WFA reaction was strong but differences were shown in the staining intensity among distinct glomeruli. Within the glomeruli, irregular islands of darker, lighter or even unstained patches were observed. The periglomerular area was very weak for WFA reaction (Fig. 2c, d). In the external plexiform layer, the superficial part was lighter, almost unstained, than its deep part (Fig. 2a–c). The WFA reaction was strong in the internal plexiform layer (Fig. 2a, b, e–g), where heavily stained bands, with lighter intervals, were shown running perpendicular to the surface of the OB (Fig. 2b). Antineurofilament labeling revealed immunoreactivity within these bands which may represent the axons of mitral cells, tufted cells, bulbar interneurons or can belong to axons from centrifugal fibers (Kosaka and Kosaka 2003). The serial pictures on Fig. 2e–g shows an example for the WFA associated mitral cell axon where the WFA staining is continued to the mitral cell layer and forms PNN around the somata of a mitral cell. The WFA reaction was weak throughout the granular layer.

**Fig. 3:**
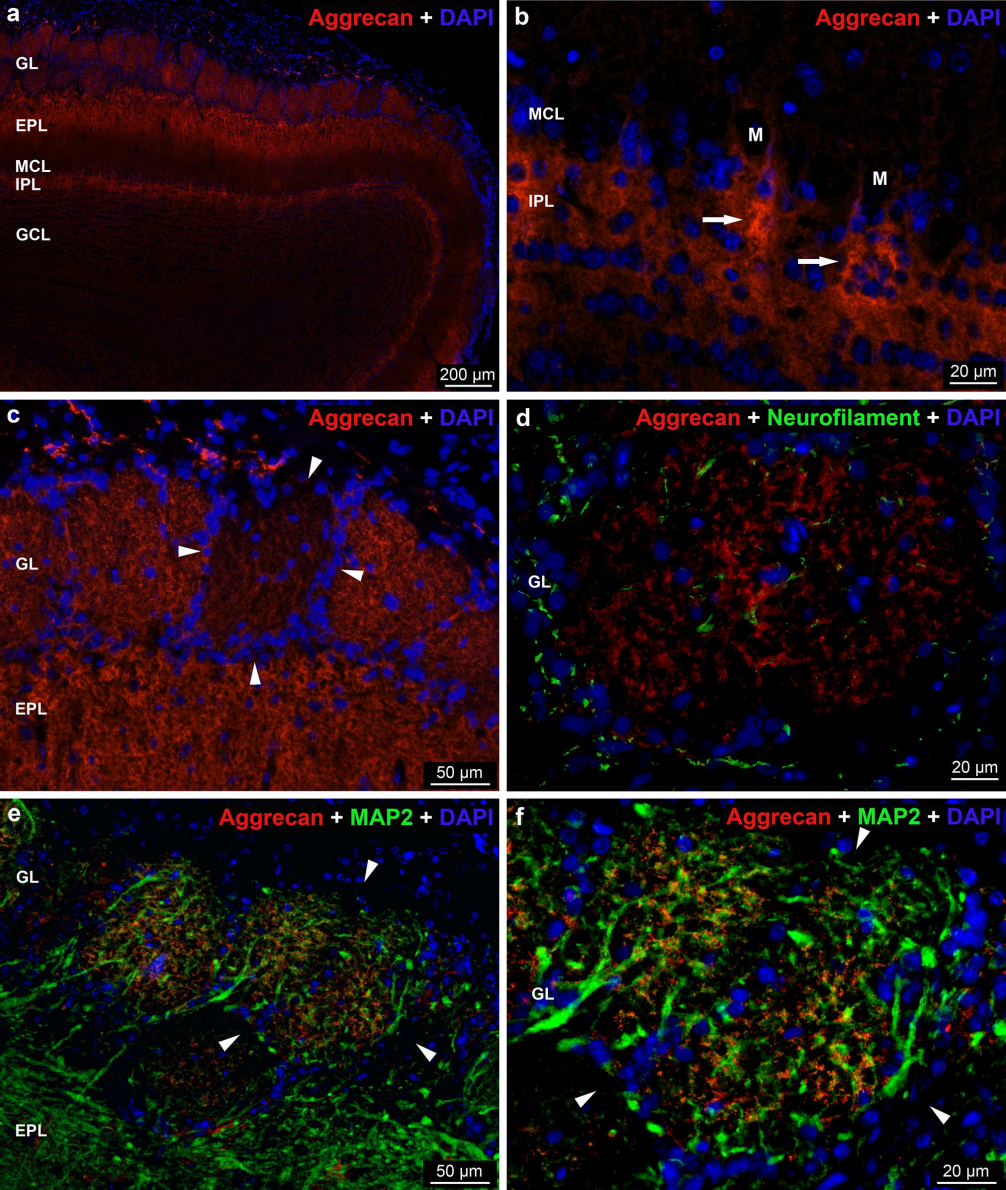
Distribution of *aggrecan* immunoreactivity in the rat OB. **a** Low magnification image showing aggrecan labeling in the layers of OB. **b** Note the intensely stained aggrecan positive bands (arrows) in the internal plexiform layer (IPL). M: cell body of mitral cells surrounded by perineuronal net. **c** Arrowheads show the weak aggrecan reaction in the periglomerular area. **d** Double labeling with aggrecan (red) and neurofilament (green). Neurofilament detects the axons in the glomeruli and periglomerular area. **e**, **f** Double immunostaining with aggrecan (red) and MAP2 (green). MAP2 labels dendrites. Arrowheads label the periglomerular area. Nuclei were stained with DAPI (**a**–**f**)

**Fig. 4:**
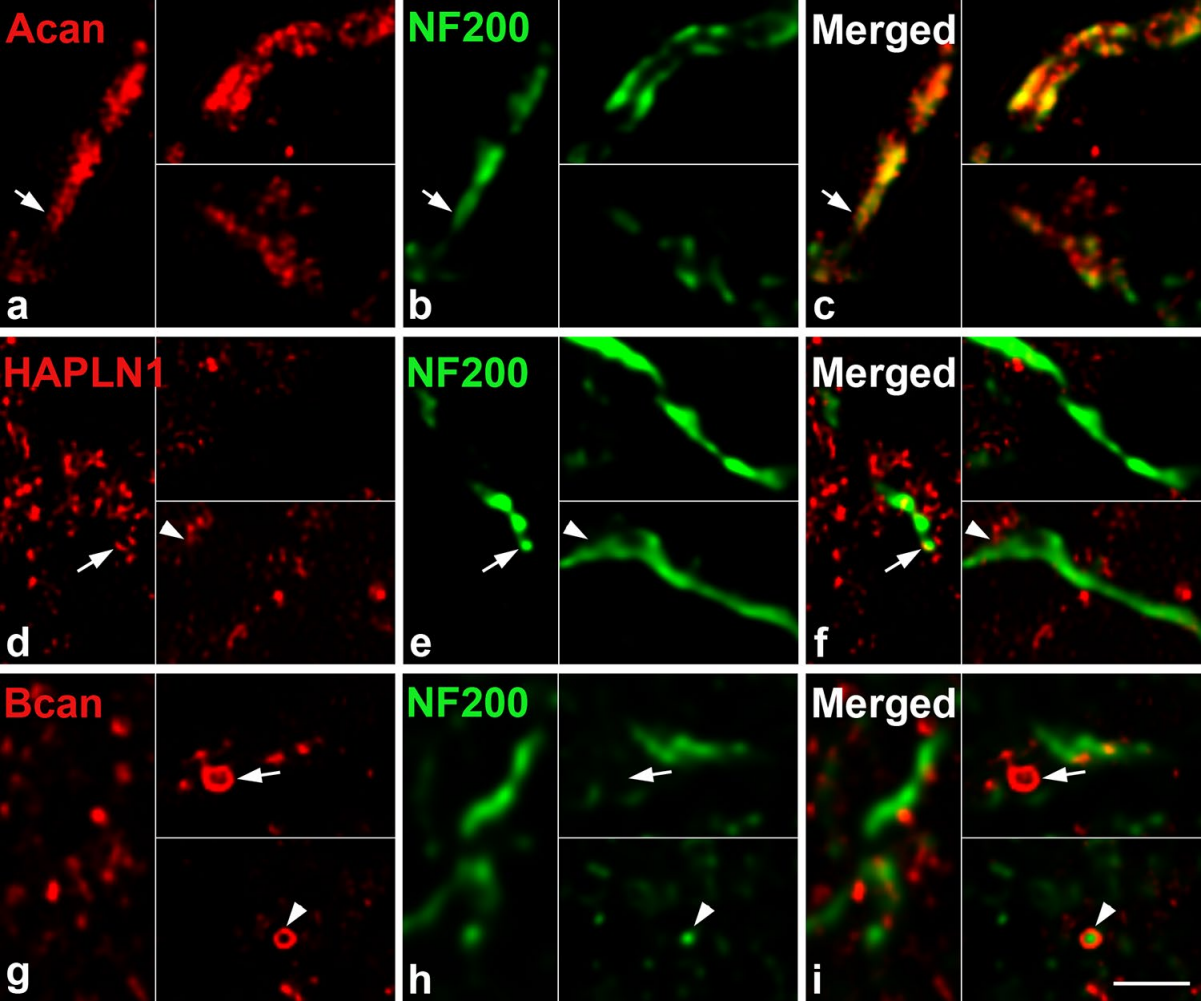
Extracellular macromolecules accumulate around the small nerve fibers in the olfactory glomeruli. Single planes of super-resolution images show the aggrecan (**a**), link protein HAPLN1 (**d**) and the brevican (**g**) immunoreaction around 200 kDa neurofilament positive nerve fibers (**b**, **e**, **h**) inside the glomerulus. Among the three extracellular macromolecules, aggrecan was the most abundant and coated the fine neurofilament positive axons with a lattice-like manner (**a**–**c**, arrows). HAPLN1 reaction was more scattered and found both inside (**d**–**f**, arrows) and close apposition with (**d**–**f**, arrowheads) the labelled axons. Brevican showed similar distribution pattern to the HAPLN1 with notable difference (**g**–**i**); brevican formed immunopositive rings around neurofilament negative structures (arrow) and positive nerve fibers (arrowheads). Scale bar: 1 µm

The *aggrecan* reaction revealed heterogeneous distribution and staining intensity in the OB (Fig. 3a). The reaction was characteristic for the neuropil. Similarly to the WFA staining, the glomerular layer showed strong aggrecan immunoreactivity in the overwhelming majority of the glomeruli, where stained and unstained patches were recognizable. Axonal coats were frequently shown in the glomeruli (Fig. 4c). To reveal whether the aggrecan staining is associated with neuronal elements, we combined the aggrecan with MAP2 or neurofilament antibodies to detect the dendrites or axons, respectively. The MAP2 reaction revealed a large number of dendrites, whereas the neurofilament staining labeled only a few axons in the glomeruli (Fig. 3d–f). The majority of the periglomerular areas were negative for the aggrecan staining (Fig. 3a, c, d). The external plexiform layer was heavily labeled with aggrecan antibody in its outer part (Fig. 3a, c). In the internal plexiform layer, aggrecan positive darker bands appeared and some of them extended into the mitral cell layer to form thin PNNs (Fig. 3b). The granular layer did not show labeling with aggrecan reaction (Fig. 3a).

**Fig. 5:**
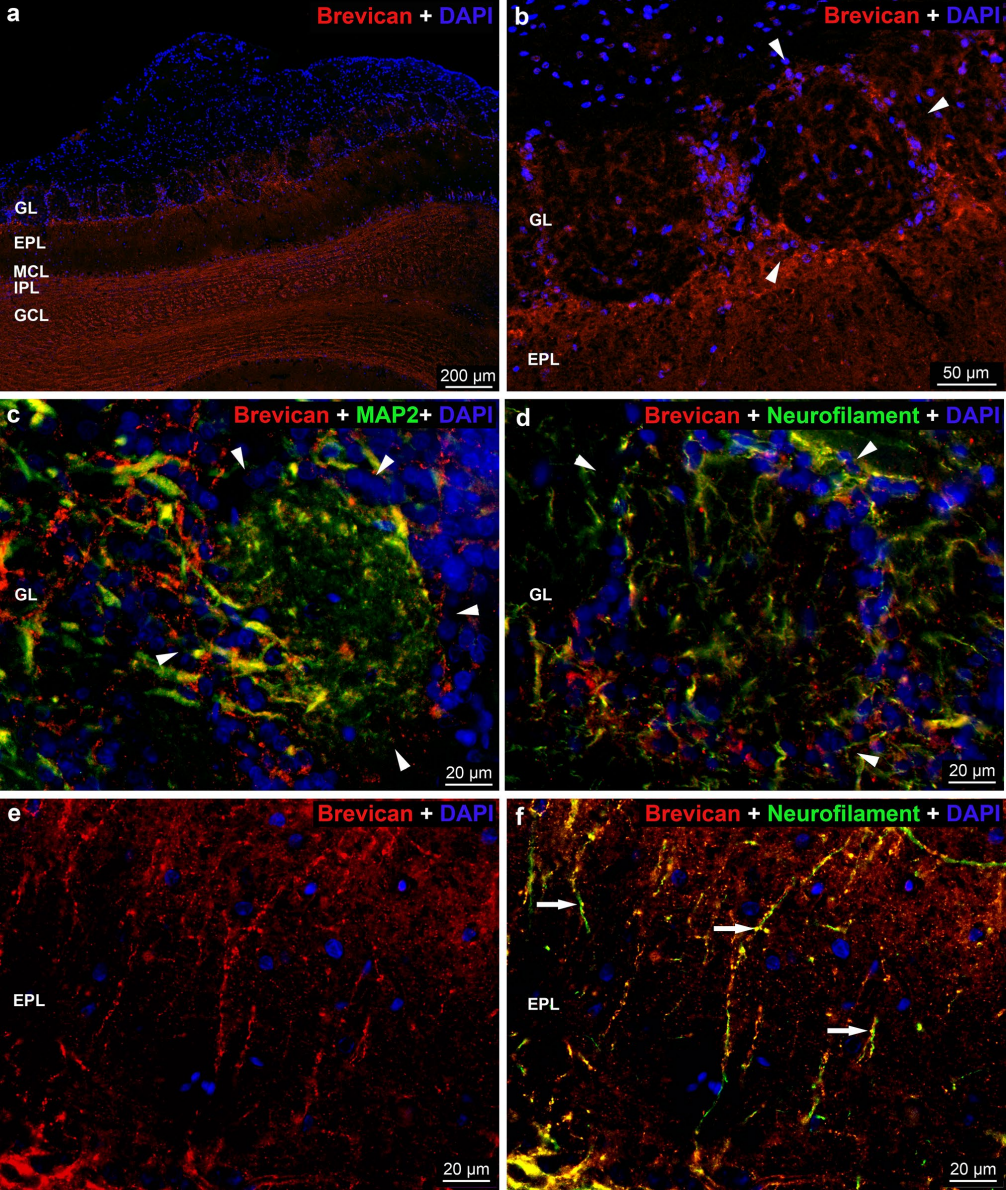
Distribution of *brevican* immunoreactivity in the rat OB. **a** Low magnification image showing brevican labeling in the layers of OB. **b** Arrowheads show the brevican positivity in the periglomerular area. **c** Arrowheads show the periglomerular area. MAP2 positivity indicates the intraglomerular dendrites. **d** Neurofilament immunoreactivity (green) detects the axons. Arrowheads show the periglomerular area. **e** In the external plexiform layer (EPL), the brevican positive beaded structures (red) are visible. **f** In the EPL, the brevican positive beaded structures (red) colocalize (arrows) with the neurofilament reaction (green). Nuclei were stained with DAPI (**a**–**f**)

In contrast to the WFA and aggrecan reactions, the *brevican* staining was weak in the glomeruli where brevican positive and negative regions (Fig. 5a, b) and axonal coats were observed (Fig. 4i). The strong MAP2 staining indicates a large number of intraglomerular dendrites, whereas the neurofilament reaction revealed much smaller number of axons (Fig. 5c, d). In the majority of the periglomerular areas the brevican staining was strong (Fig. 5a, b). In the uppermost part of the external plexiform layer a narrow strip was intensely stained whereas the rest of the layer showed weak staining with brevican antibody (Fig. 5a). Higher magnification revealed brevican positive beaded structures running perpendicular to the surface of the OB (Fig. 5e). Double-labeling experiments showed that these beads colocalize with the neurofilament reaction suggesting the nodal form of ECM along the axons containing brevican molecules (Fig. 5e, f). Most of the mitral cells were surrounded by thin perineuronal net (not shown). The internal plexiform and granular layers of the OB showed intense brevican reaction without regional differences within the layers (Fig. 5a).

**Fig. 6:**
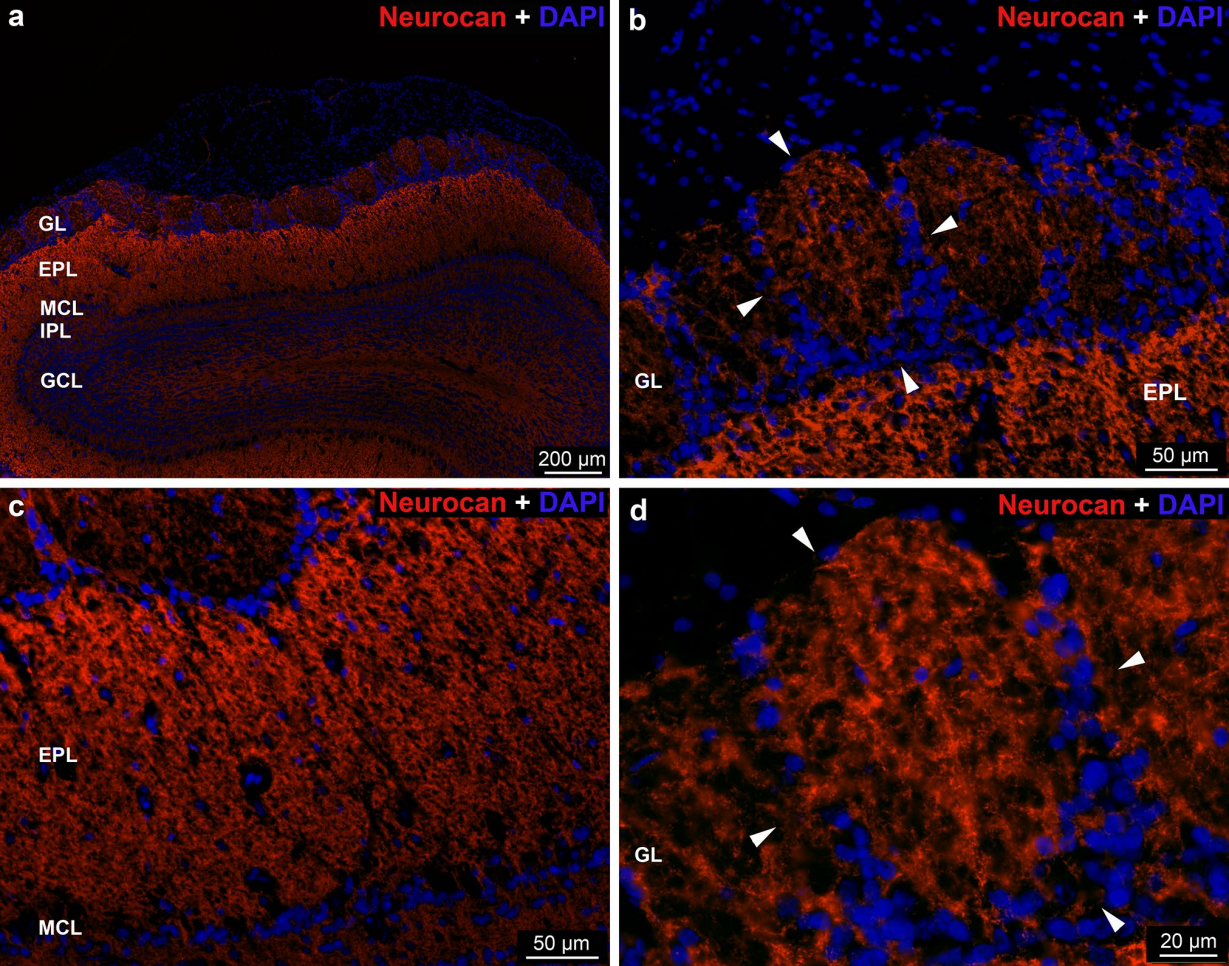
Distribution of *neurocan* immunoreactivity in the rat OB. **a** Low magnification image showing neurocan labeling in the layers of OB. **b**, **d** Arrowheads show the periglomerular area. Nuclei were stained with DAPI (**a–d**)

The *neurocan* reaction was detected throughout the OB (Fig. 6a). In the glomerular layer, positive and negative areas were shown within the glomeruli, no differences were recognizable between the staining intensity of the glomeruli. The staining intensity of the periglomerular area was moderate (Fig. 6b, d). The external plexiform layer displayed the strongest reaction, especially in the outermost part (Fig. 6b, c). The internal plexiform layer showed moderate neurocan immunoreactivity which was weak in the granular layer (Fig. 6a).

**Fig. 7:**
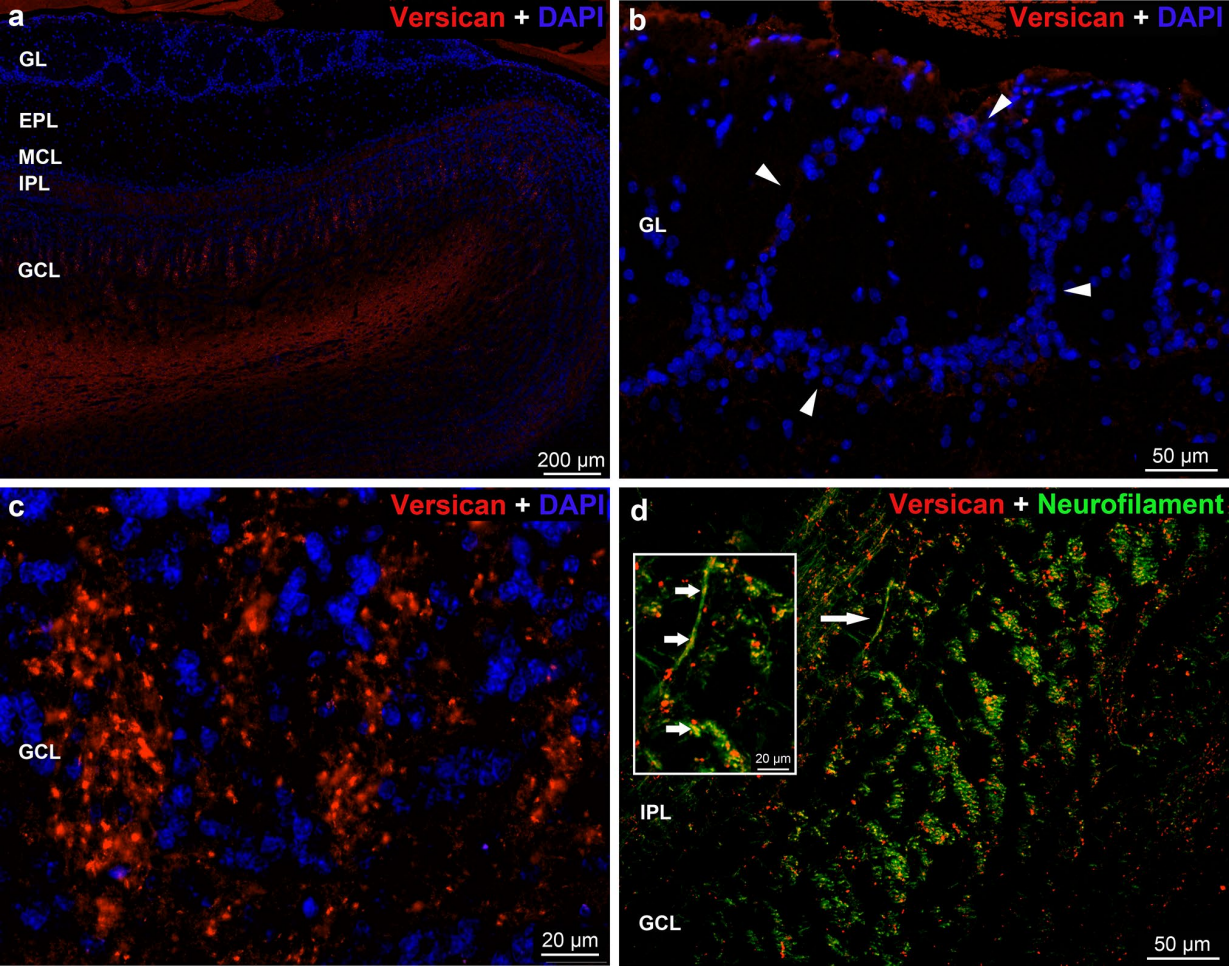
Distribution of *versican* immunoreactivity in the rat OB. **a** Low magnification image showing versican labeling in the layers of OB. **b** Arrowheads show the periglomerular cells. Note the columnar organization of versican positive dots in **a**, **c** and **d** panels. **d** Overlap of neurofilament staining with the versican positive dots (arrow) on both low- and high magnified images. Nuclei were stained with DAPI (**a–d**)

The *versican* staining was almost negative in the glomerular- and external plexiform layer as well as in the mitral cell layer (Fig. 7a). In the internal plexiform and granular layers (Fig. 7a, c, d) characteristic dot-like appearance of the versican reaction (Bekku et al. 2009) was detected. They were present in higher number in the outer part of the granular layer where alternating darker and lighter columns were shown due to the higher or lower number of versican positive dots, respectively (Fig. 7a, c). Neurofilament co-labeling revealed a similar columnar organization; the versican positivity showed a partial overlap with the neurofilament staining in the granular layer (Fig. 7d). In the inner part of the granular layer, versican positive dots were present in lower density and here the neurofilament staining was very weak.

**Fig. 8:**
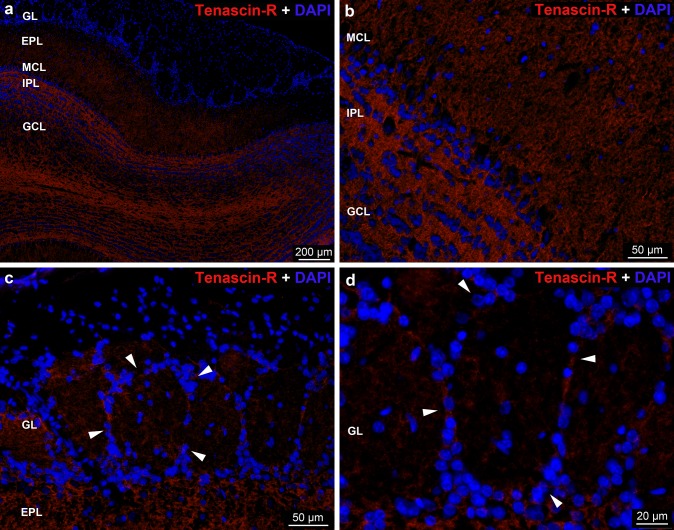
Distribution of *tenascin-R* immunoreactivity in the rat OB. **a** Low magnification image showing tenascin-R labeling in the layers of OB. **b** Uneven distribution of tenascin-R in the MCL, IPL and GCL of OB. **c**, **d** Arrowheads show the periglomerular area. Nuclei were stained with DAPI (**a**–**d**)

The staining intensity of *TN-R* reaction varied in the OB (Fig. 8a). The reaction was almost negative in the entire glomerular layer both in and around the glomeruli (Fig. 8a, c, d). The strongest reaction was visible in the internal plexiform layer, whereas the external plexiform and granular cell layers showed moderate staining (Fig. 8a, b). Immunoreactivity was restricted to the neuropil, the PNNs, nodal ECM and axonal coats were not detected with TN-R staining.

**Fig. 9:**
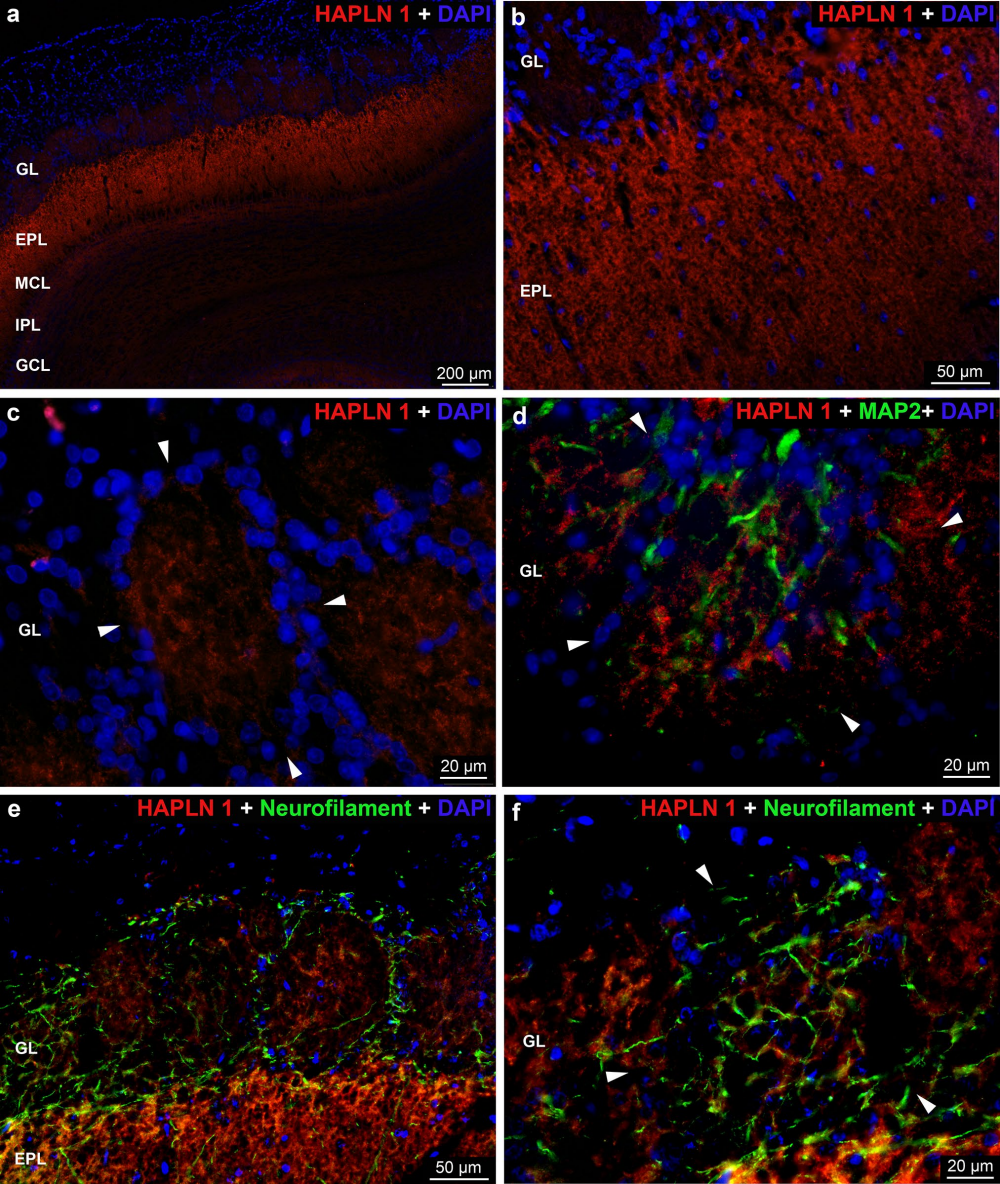
Distribution of *HAPLN1* immunoreactivity in the rat OB. **a** Low magnification image showing HAPLN1 labeling in the layers of OB. **b** Uneven distribution of HAPLN1 in the GL and EPL of OB. **d**–**f** MAP2 and neurofilament positive areas represent the dendrites and axons, respectively. **c**, **d**, **f** The arrowheads indicate the border of periglomerular area. Nuclei were stained with DAPI (**a–f**)

The *HAPLN1* reaction was present in the glomerular layer showing the uneven distribution and variable staining intensity between the glomeruli and within the individual glomeruli (Fig. 9a, c). The MAP2 and neurofilament antibodies showed positive reaction among the HAPLN1 stained areas (Fig. 9d–f). Higher magnification revealed axonal coats in the glomeruli (Fig. 4f). Very week HAPLN1 reaction was detected in the periglomerular area (Fig. 9c–f). The strongest staining was present throughout the external plexiform layer, followed by the moderate staining intensity in the internal plexiform layer. The granular cell layer was negative for HAPLN1 staining (Fig. 9a, b).

### Distribution pattern of ECM in the olfactory bulb of wild type and KO mice

We investigated if typical chondroitin sulphate proteoglycan components were expressed in the olfactory bulb of wild type C57/Bl6 mice (WT NCAN) and if these patterns are altered in their homozygous neurocan knockout littermates (NCAN KO).

**Fig. 10:**
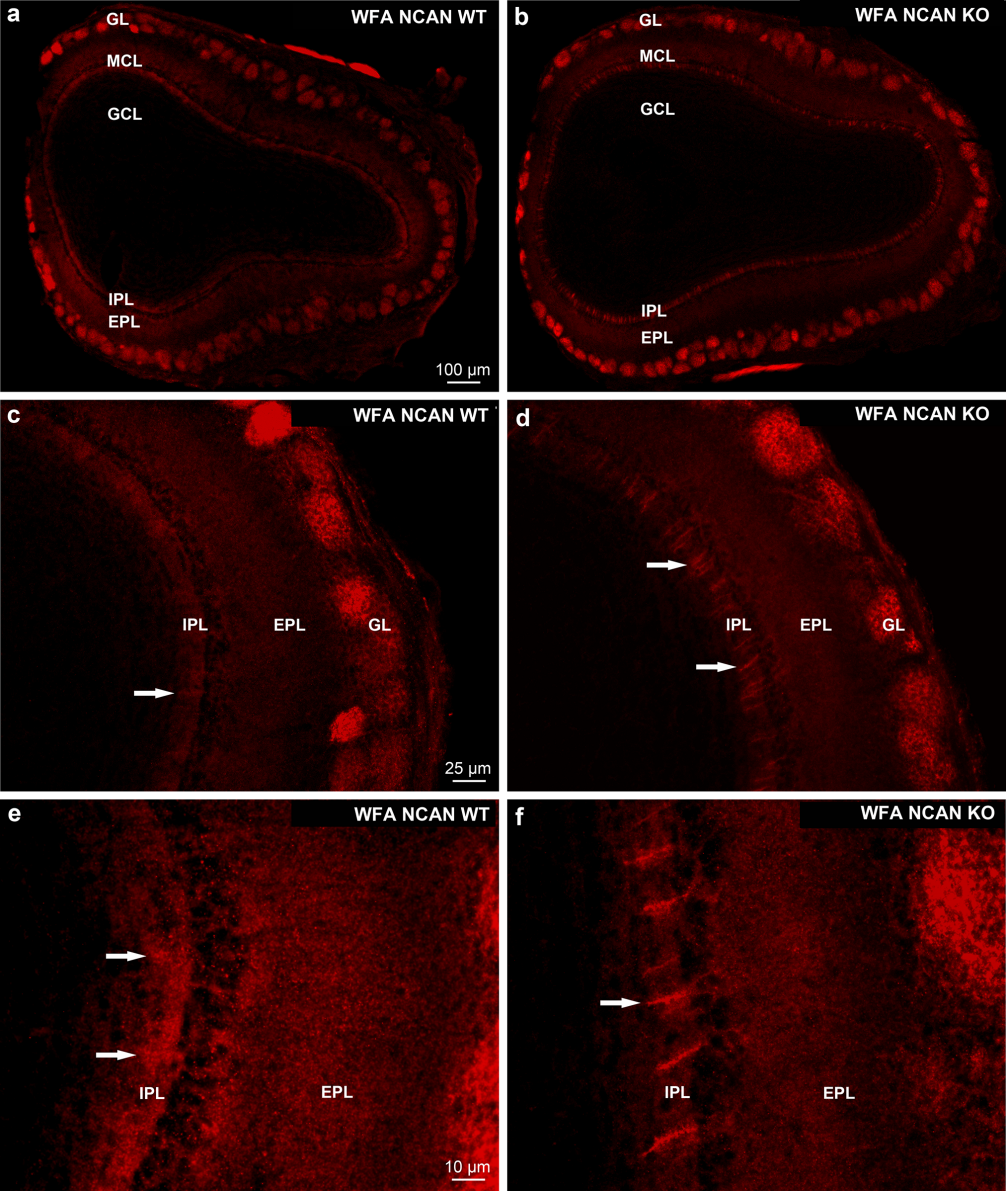
Distribution of *Wisteria floribunda* agglutinin (WFA) reaction in wild type C57/Bl6 mice (NCAN WT*)* and neurocan knockout littermates (NCAN KO) in the OB. **a**, **b** Layer specific proteoglycan distribution was shown with WFA staining. **c**, **e** White arrows in the outer part of the IPL of NCAN WT show stripped proteoglycan accumulations in diffuse appearance. In the NCAN KO littermates, however, stripes show distinct staining pattern (**d**, **f**)

In wild type (WT NCAN) mice the WFA reaction was detected in the neuropil of the whole OB, showing layer-specific staining pattern and intensity (Fig. 10a, c, e) comparable to the rat OB (Fig. 2a–d). In the glomerular layer, the WFA reaction was strong and differences were shown among distinct glomeruli regarding the intensity of staining (Fig. 10a–d). Comparing these WT patterns with the patterns of NCAN KO littermates the most compelling difference is the change in the IPL from a diffuse appearance (Fig. 2c, e; white arrows) of WFA staining of the WT mice to a very defined stripe-like appearance (Fig. 2d, f; white arrows) in the NCAN KO littermates.

**Fig. 11:**
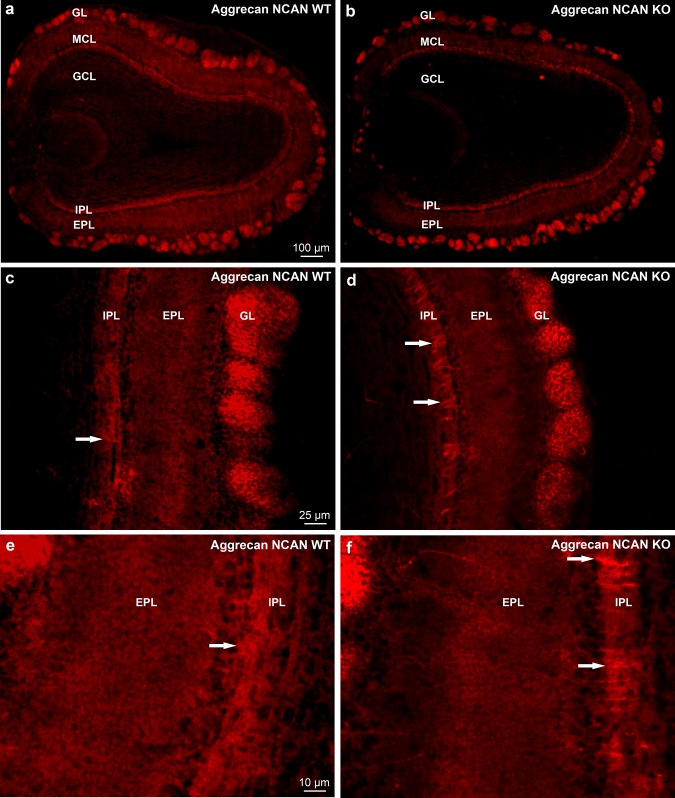
Distribution of *aggrecan* immunoreactivity in wild type C57/Bl6 mice (NCAN WT; **a**, **c**, **e**) and neurocan knockout littermates (NCAN KO; **b**, **d**, **f**) in the OB. **a**, **b** Note the layer-specific staining of aggrecan in low magnification. **c**–**f** Arrows point a clear differences between the WT and NCAN KO littermates regarding the diffuse appearance of transversed stripped aggrecan accumulations (**c**, **e**), in strong contrast with the defined stripe-like appearance (**d**, **f**) in the IPL

The aggrecan immunoreaction in mice revealed also a heterogeneous distribution and staining intensity in the OB (Fig. 11a–d) comparable to the rat OB (Fig. 3a). Aggrecan reaction was characteristic for the neuropil. Similarly to the WFA staining, the glomerular layer showed strong aggrecan immunoreactivity in the majority of the glomeruli, where strongly stained and virtually unstained glomeruli were recognizable. Again, comparing these WT patterns with the patterns of NCAN KO littermates the most compelling difference is the change in the IPL from a diffuse appearance (Fig. 11c, d; white arrows) of aggrecan staining of the WT mice to a very defined stripe-like appearance (Fig. 11e, f; white arrows) in the NCAN KO littermates. The majority of the periglomerular areas were negative for the aggrecan staining (Fig. 11c, d).

**Fig. 12:**
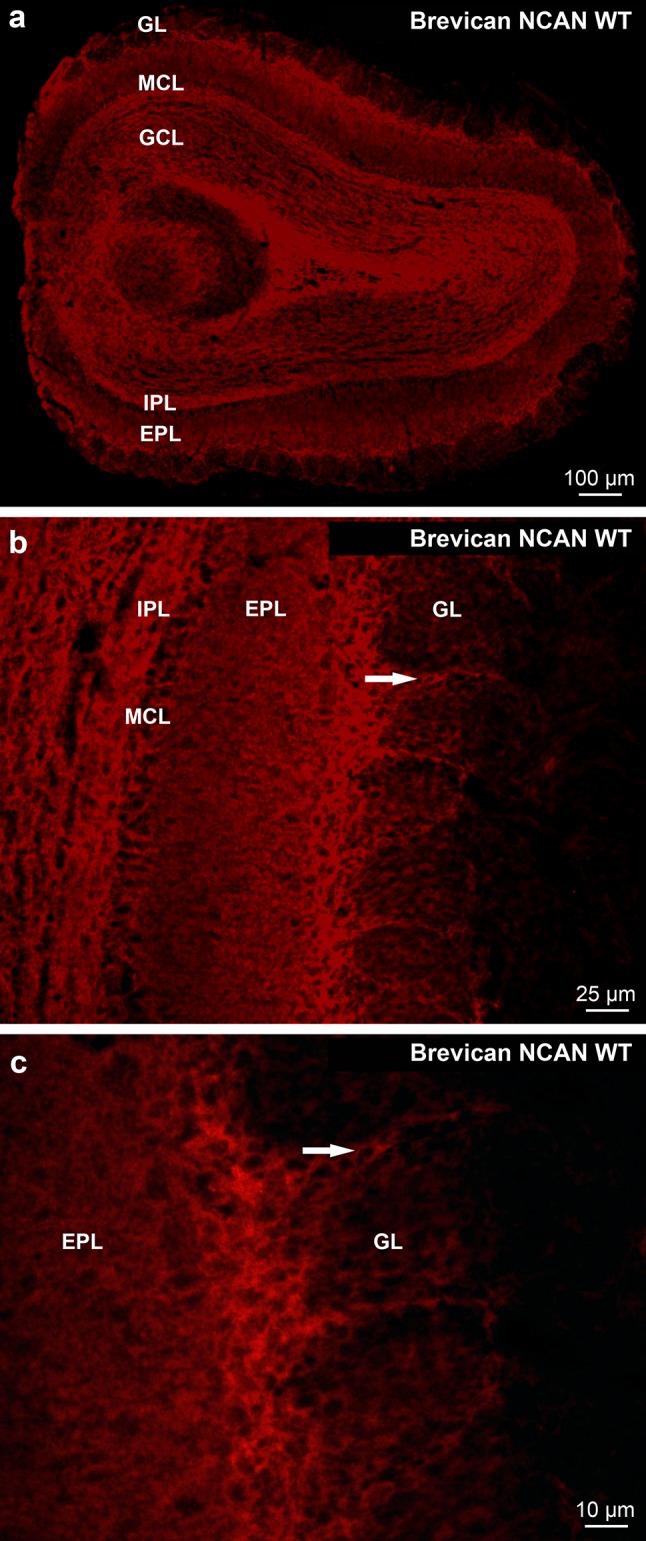
Distribution of *brevican* immunoreactivity in wild type C57/Bl6 mice (NCAN WT; **a**–**c**). **b**, **c** Strong brevican positivity was shown in the periglomerular area (arrows) and in the outer part of the EPL, pericellularly in the mitral cell layer (MCL) and in the IPL

Also in mice brevican immunoreaction was weaker (Fig. 12a) in contrast to the WFA and aggrecan reactions. Brevican staining was very weak in the glomeruli (Fig. 12). However, brevican positive and negative regions (Fig. 12) and axonal coats could sometimes be observed. In the majority of the periglomerular areas brevican staining was strong (Fig. 12b, c). In the uppermost part of the EPL a strip was intensely stained also in mice (Fig. 12b, c; white arrows) like in the rat OB (Fig. 5a, b). No difference was found comparing these WT patterns with the patterns of NCAN KO littermates.

### Distribution pattern of ECM in the olfactory bulb of human

**Fig. 13:**
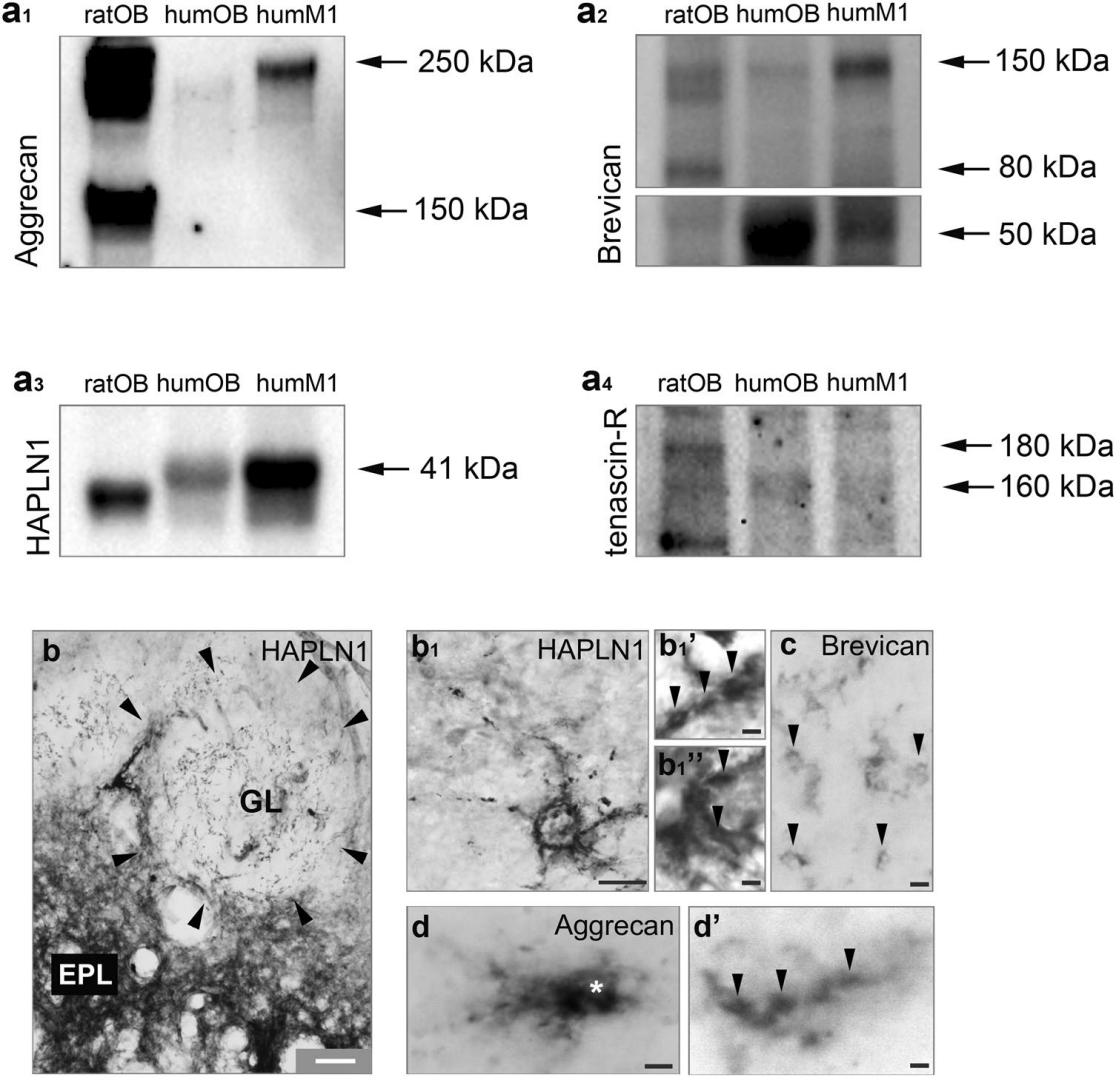
**a**_**1**_**–**_**4**_ Representative Western blots of aggrecan, brevican, HAPLN1 and tenascin-R expression in the rat (ratOB) and human olfactory bulb (humOB) and in the human primary motor cortex (humM1). **b** HAPLN1 immunoreactivity in the human olfactory bulb. Glomeruli (marked with arrowheads) appear as lightly stained structures. (**b**_**1**_**–**_**1**_”) HAPLN1 immunoreactivite structures were typically identified as axonal coats (arrowheads). **c** Brevican-immunoreactive axonal coats in glomeruli (arrowheads). **d**, **d’** Aggrecan-immunoreactive perineuronal net (asterisk indicates soma) and axonal coats (arrowheads). Scale bars 150 μm (**b**), 10 μm (**b**_**1**_, **d**), 3 μm (**b**_**1**_’, **b**_**1**_”, **c**, **d**’)

Finally, we also investigated if typical chondroitin sulphate proteoglycan components were expressed in the human olfactory bulb. Humans are microsmatic creatures with an olfactory bulb of a relatively reduced size. We first used Western blotting to show the presence of extracellular matrix components in human and rat brains in parallel (Fig. 13a1–4). Compared to rat olfactory bulb tissue we could detect only weak aggrecan expression at the 250 kDa molecular weight using the AB1031 antibody in the human olfactory bulb (Fig. 13a1). Brevican was well detectable, especially its 50 kDa fragment (Fig. 13a2), as well as HAPLN1 at the 41 kDa molecular weight (Fig. 13a3). Tenascin-R expression could be detected at the 160 kDa molecular weight level (Fig. 13a4).

We then used immunohistochemistry to explore the phenotype of the above extracellular matrix component-immunoreactive structures. Extracellular matrix components showed a distribution pattern which was similar we found in the murine olfactory bulb (Hanics et al. 2017). HAPLN-1 immunoreactivity was concentrated in the external plexiform layer with the glomerular layer being rather spared from immunostaining (Fig. 13b). Of note, HAPLN1 labelled delicate structures within the otherwise immunonegative glomerulus in high density (Fig. 13b1) which appeared as small ring-like structures (Fig. 13b1, b1’, b1”). These axonal coat-like structures were also visible anti-brevican immunohistochemistry (Fig. 13c). In addition to aggrecan-containing diffuse perineuronal nets (Fig. 13d), axonal coat-like structures could also be identified by anti-aggrecan immunohistochemistry (Fig. 13d’).

## Discussion

Extracellular matrix became an important player over the last few decades when studying the plasticity and regeneration of the central nervous system. In spite of the established role of ECM in these processes throughout the CNS, only few papers were published on the ECM of the olfactory system, which shows a lifelong plasticity, synaptic remodeling and postnatal neurogenesis. In the present study, we have described the localization and organization of major ECM molecules, the hyaluronan, the lecticans, tenascin-R and HAPLN1 link protein in the olfactory bulb of the rat. Using histochemical and immunohistochemical methods, we detected all of these molecules in the OB showing differences in the molecular composition, staining intensity and organization of ECM between the layers and in some cases within a single layer. We found both the diffuse and condensed forms of ECM. In most cases, the staining was detected in the neuropil throughout the olfactory bulb. Axonal coats and nodal ECM were also recognizable, however, perineuronal nets were rarely present in the OB. In some parts of discussion, we compare the results of rat with mice and human specimens.

### Distribution of ECM in layers of the olfactory bulb

In the *glomerular layer*, each ECM reaction, except the versican, was positive, however staining intensities varied in case of different reactions. Overall, the glomeruli showed the most intense staining with WFA and aggrecan reactions followed by moderate hyaluronan, neurocan and HAPLN1 staining. The immunoreactivity was the weakest with brevican and it was almost negative with TN-R antibodies. Since the versican and TN-R are essential components of the nodal ECM (Bekku et al. 2009) the absence of versican and the very weak TN-R reaction may be related to the absence of Ranvier nodes in the glomeruli due to the presence of nonmyelinated olfactory nerve axons (Garcia-Gonzalez et al. 2013). A characteristic feature of the layer was the mixture of strongly and weakly stained glomeruli shown with WFA, aggrecan and HAPLN1 reactions. The interpretation of these findings needs further studies. In the glomeruli, the other characteristics of the ECM reactions was an inhomogeneous staining, showing irregularly stained and unstained areas. On the basis of our results, we can merely state that this staining pattern resembles to the two compartments of the olfactory glomeruli (Kosaka et al. 1997, 1998). The olfactory nerve compartment is the zone where the axon terminals establish synaptic contacts with the intrinsic neurons, whereas the non-olfactory compartment is the place of the dendro-dendritic contacts of the intrinsic- and projection neurons. Further experiments, e.g., combination of ECM stainings with the specific markers of the olfactory nerve and non-olfactory nerve compartments are needed to show a possible overlap.

The unequal distribution of ECM staining in the glomeruli may suggest that a given ECM molecule contributes differently to the synaptic plasticity in the two compartments of the glomeruli. This suggestion is strengthened by the different expression dynamics of the aggrecan and brevican regarding their different mRNA copy number and turnover (Milev et al. 1998; Zimmermann and Dours-Zimmerman 2008). In the glomeruli, the condensed ECM was represented with the axonal coats shown by WFA, aggrecan, brevican and HAPLN1 reactions.

Staining pattern in the periglomerular areas was also characteristic for the individual reactions. The strongest staining was shown with HA and brevican reactions., it was moderate with the neurocan antibody but the staining remained weak when surveying WFA-lectin reactivity or aggrecan, neurocan- and TN-R immunoreactivity. Further, we were unable to detect versican and HAPLN1-expressing matrix in this area. The variable expression pattern of ECM molecules supposed to be related to the morphological, functional and neurochemical characteristics of the periglomerular cells. The strong expression of the HA might be associated with the long-lasting immature properties of calretinin expressing periglomerular cells (CR-PG). Benito et al. (2018) showed that unlike other postnatally generated-newborn neurons, the recruitment of CR-PG cells into the existing local network is limited and they may serve as a reserve pool for a functional maturation or may differentiate into other types of periglomerular cells. On the other hand, the HA rich milieu is known to promote the neural migration, axonal sprouting, the maturation of parvalbumin-positive cells and controls synapse plasticity (Margolis et al. 1975; Miyata and Kitagawa, 2017; Preston and Sherman 2011; Wakao et al. 2011). Furthermore, on the basis of data on the other parts of CNS cited below, it is temting to assume that the strong expression of brevican may control the synaptic plasticity in the already established synapses in the periglomerular region. The perisynaptically located brevican limits the lateral diffusion of AMPA-receptors which reduces the exchange of synaptic and extrasynaptic receptors in the adult brain which might be important for the functionality of mature synapses (Frischknecht et al. 2009; Seidenbecher et al. 1997). The brevican simultaneously controls cellular and synaptic forms of plasticity in parvalbumin-positive cells by regulating the localization of potassium channels and AMPA receptors, respectively (Favuzzi et al. 2017). Recently, the contribution of brevican in the spatial coupling of pre- and postsynaptic elements was experimentally established in the cochlea as an important precondition for the ultrafast synaptic transmission (Sonntag et al. 2018). The time course of brevican expression correlates with this finding as it begins to appear during the first postnatal week, and its expression reaches a maximum in adult rat (Milev et al. 1998; Seidenbecher et al. 1998; Zimmermann and Dours-Zimmermann 2008). We suppose that the described functions of HA and brevican may be applied to the other layers of the olfactory bulb. The presence of PNNs cannot be excluded in the periglomerular area, but the densely packed cells did not allow to identify the pericellular aggregation of the ECM molecules.

All the ECM reactions studied, except versican, were positive in the *external plexiform layer*. The overall staining intensity was strongest with the HA, aggrecan, neurocan and HAPLN1, followed by moderate WFA and brevican reactions and weak TN-R immunoreactivity. The intensity of reactions was almost homogeneous throughout the layer with HAPLN1. In other cases the lighter and darker zones, running parallel with the surface of the OB, seemed to be correlated with the two sublayers of EPL. The EPL is conventionally divided into an outer/superficial and inner/deep parts (Mizuguchi et al. 2012; Mori et al. 1983), on the basis of differences in the position of mitral- and tufted cell somata and territories of their secondary dendrites (Mori et al. 1983; Nagayama et al. 2014; Orona et al. 1984). Given the experimental data that the secondary dendrites of mitral and tufted cells establish different synaptic contacts in the different sublayers (Nagayama et al. 2014) it is tempting to assume that the unequal distribution of ECM molecules provides a special microenvironment for the local synaptic circuits. We found that the HA, aggrecan, brevican and neurocan reactions were more intense in the outer part of the external plexiform layer. At this point, we have commented the role of HA and brevican on the maturation and involvement of parvalbumin-positive cells in neural plasticity to refer to page 15. Although the parvalbumin-positive neurons are found throughout the olfactory bulb, the highest expression was shown in the outer part of the external plexiform layer (Kosaka et al. 1994, 1995; Kosaka and Kosaka 208; Liberia et al. 2013).

Interestingly, the localization of WFA reaction was inverse to those of the aggrecan staining. Thus, the aggrecan staining was strong in the outer, whereas the WFA reaction was intense in the deep part of the external plexiform layer. This result is in contrast to the previous finding that the WFA staining is mostly dependent on the presence of aggrecan in mammals and most likely specific for its *N*-acetylgalactosamin carbohydrate epitopes (Giamanco et al. 2010). At present, we cannot give an explanation for this contradiction. The ECM reactions did not reveal an intermediate sublayer in the external plexiform layer, however the very strong brevican positive zone underneath the glomeruli, where the cell bodies of the secondary dendrite-bearing external tufted cells are located, may suggest an ECM-based sublayer in the external granular layer. Based on the intense cytochrome oxidase staining, an intermediate zone was also distinguished between the faintly stained superficial and deep zones (Mouradian and Scott 1988). In the external plexiform layer, the neurocan staining was strong. This molecule is an essential component of juvenile ECM and its level is dramatically decreased during the early postnatal life in the brain extract (Milev et al. 1998; Zimmermann and Dours-Zimmermann 2008). The persistent expression of neurocan in the olfactory bulb is consistent with the high degree of plasticity in the olfactory system. Among the lecticans, the brevican antibody gave a characteristic beaded appearance. Its colocalization with the neurofilament reaction may indicate that the large amount of brevican molecules is located at the nodes of Ranvier.

The *internal plexiform layer* is the only one which showed positivity with each ECM reaction studied. The layer is populated mostly by axons, the few cell bodies represent one of the subtypes of deep short-axon cells, the GL-dSA (Eyre et al. 2008; Nagayama et al. 2014). One of the inputs to the layer arrives from the opposite side of the same olfactory bulb, the external tufted cells establish excitatory synaptic contacts predominantly on the secondary dendrites of GABAergic GL-dSA along with a smaller contingent of the apical dendrites of EPL- and GCL-dSA neurons, and type II and IV granule cells (Belluscio et al. 2002; Burton et al. 2017; Liu and Shipley 1994; Lodovichi et al. 2003). The connection is mirror-symmetric and the established “intrabulbar map” may allow the two halves of the OB to coordinate the activity-dependent modification of the map (Cummings and Belluscio 2008, 2010). The activity of the intrabulbar map can be modified by the axons of horizontal diagonal band of Broca (HBD) of the basal forebrain which terminate on the GL-dSA (Price and Powell 1970). The excitatory input is provided by the cholinergic and glutametergic neurons of HBD, some of the terminals release both acetylcholine and GABA (Case et al. 2017). In the lack of detailed knowledge on the precise function of particular ECM molecules in the synaptic transmission and plasticity we do not know the reason of their strong expression in the IPL. We can only hypothesize that is associated with the high degree of plasticity in the “intrabulbar map”. It can be supported by the changing of WFA and aggrecan staining pattern in the IPL in mice. We have found that their diffuse appearance in the WT mice is changing to a very defined stripe-like appearance in the NCAN KO littermates. Among the other molecules studied, the tenascin-R deserves further discussion. The TN-R expression was the highest in the internal plexiform, moderate in the granular layer, whereas the immunoreactivity was very weak or negative in the superficial layers of OB. Similar distribution was described in adult mouse (David et al. 2013; Saghatelyan et al. 2004) and important role of TN-R was established in OB neurogenesis. These observations showed that the TN-R induces the radial migration of newly formed neuroblasts into the OB and increases the spine density on the dendrites of newborn neurons. The stronger expression of TN-R in the deeper parts of the OB correlate with the direction of radial migratory pathway and the finding that the majority of newborn neurons differentiate into granular and periglomerular cells (Sakamoto et al. 2014a, b). On the other hand, the tenascin-R regulates the GABA_B_ receptor-mediated perisomatic inhibition and thus influences synaptic transmission and plasticity in the hippocampus (Brenneke et al. 2004; Bukalo et al. 2001; Dityatev and Schachner 2003; Saghatelyan et al. 2000, 2001). Although the type of GABA receptor in the internal plexiform layer was not yet determined, similar function of the TN-R cannot be excluded.

The *granule cell layer* showed the most intense immunostaining with brevican reaction followed by moderate the HA, neurocan, TN-R reactions, and weak WFA staining. The aggrecan and HAPLN1 antibodies did not label the layer. The intensity of these stainings was uniform throughout the layer. The strong brevican reaction and the negative aggrecan staining indicate that the main ECM component of the perisynaptic ECM is the brevican as it was shown in the basket-like synapses of the inner hair cells of the cochlea (Sonntag et al. 2018). The most characteristic staining pattern was the columnar organization of versican positive dots representing the presence of this ECM molecule at the nodes of Ranvier. The dots were much more numerous in the superficial half of the layer.

### Functional considerations

One of the striking features of ECM staining pattern in the OB is that the reactions are shown dominantly in the neuropil. PNNs were present only in the mitral cell layer with the WFA and aggrecan staining and they exhibited only the thin appearance, other forms were not recognizable. These results are in agreement with the life-long plasticity of the olfactory system which includes formation and elimination of synaptic contacts and continuous generation and migration of interneurons into the OB (Imai 2014). The PNNs limit the plasticity in adulthood by altering new neuronal contacts, acting as a scaffold for molecules that can inhibit synapse formation, and limiting receptor motility at synapses (Barritt et al. 2006; Corvetti and Rossi 2005; Deepa et al. 2002; Frischknecht et al. 2009; Sorg et al. 2016; Wang and Fawcett 2012).

The other interesting point of our results is the comparison of the ECM expression in two compartments of the olfactory bulb which have a major role in the plasticity. One of them is in the glomeruli and called “glomerular map”, whereas the other, the “intrabulbar map” is located in the internal plexiform layer. The expression of ECM molecules is very similar in the two “maps” except the very strong TN-R expression in the IPL, which is almost negative in the glomeruli. The possible explanation may be related to different periods in the modification of the neuronal circuits in the two maps. During development, the enhancing odorant-induced activity with odorant conditioning has been shown to accelerate the refinement of both glomerular (Kerr and Belluscio 2006) and intrabulbar circuitry (Marks et al. 2006). In contrast, the long-term naris closure causes altered the glomerular refinement if it begins soon after birth (Nakatani et al. 2003; Zou et al. 2004), but it is not shown in later periods of life. Interestingly, the intrabulbar map appears extremely responsive to reductions of afferent stimulation, since olfactory deprivation, which begins either during development or adulthood, results in a broadening of intrabulbar projections (Marks et al. 2006). Thus, while the axonal projections that make up the glomerular and intrabulbar maps are changing for the altered levels of olfactory stimulation, they do not exhibit the same degree of plasticity.
